# Tailoring electromagnetically induced transparency with different coupling mechanisms

**DOI:** 10.1038/srep21457

**Published:** 2016-02-22

**Authors:** Hai-ming Li, Shao-bin Liu, Shen-yun Wang, Si-yuan Liu, Yan Hu, Hai-bin Li

**Affiliations:** 1 Key Laboratory of Radar Imaging and Microwave Photonics, Nanjing University of Aeronautics and Astronautics, Nanjing, 210016, China; 2 Research Center of Applied Electromagnetics, Nanjing University of Information Science & Technology, Nanjing, 210044, China; 3 College of Materials and Chemical Engineering, Anhui Jianzhu University, Hefei 230022, China

## Abstract

Tailoring electromagnetically induced transparency with two different coupling mechanisms has been numerically demonstrated. The results show that EIT based on simultaneous electric resonance and magnetic resonance has relatively larger coupling distance compared with that based on electric resonance near field coupling to magnetic resonance. The relatively large coupling distance is due to the relatively small susceptibility change. For EIT based on simultaneous electric resonance and magnetic resonance, not only incident electric field but also the incident magnetic field pays a role on the susceptibility of system. The influence of the incident magnetic field leads to relatively smaller susceptibility change compared with that based on electric resonance near field coupling to magnetic resonance.

Metamaterials can exhibit fascinating electromagnetic properties beyond nature materials, such as optical magnetism^1^,^2^, superlenses^3^,^4^, negative index of refraction^5^,^6^, invisibility clocking^7^,^8^, perfect electromagnetic wave absorber^9^,^10^. Therefore, tremendous attentions have been paid to metamaterials over the past decades. Metamaterials can be constructed by meta-atoms, which is similar to the solids consisting of certain kinds of atom. Electromagnetically induced transparency (EIT) is the quantum interference phenomenon in three-level atomic systems, which can be analogously re-exhibited by metamaterials. EIT in atomic systems needs harsh experimental conditions^11^, which severely limits its potential applications. However, artificial metamaterials are easy to exhibit analogy of electromagnetically induced transparency behaviors. Zhang *et al.*^12^ have theoretically exhibited that EIT can be obtained with metamaterials consisting of radiative mode and subradiant mode. Henceforth, lots of EIT^13^,^14^,^15^,^16^,^17^,^18^,^19^,^20^ have been theoretically and experimentally demonstrated.

The structure symmetry breaking^21^,^22^,^23^ and the near field subwavelength scale coupling^12^,^24^ both can induce EIT, which enrich EIT research. Zhang *et al.*^22^,^23^ have theoretically and experimentally demonstrated that EIT can be obtained by asymmetry metamaterials structure. For near field subwavelength scale coupling, most of EIT^25^,^26^,^27^,^28^,^29^,^30^ can be interpreted by the near field coupling between the bright mode and the dark mode. The bright mode can be directly excited by the incident electromagnetic wave. The dark mode has no interaction with the incident electromagnetic wave. It only can be excited by the near field coupling of the bright mode, which indicates that the coupling distance between the bright mode and the dark mode pays an import role in inducing EIT. As the distance increases, transparency window of EIT will deteriorate. When the coupling distance increases beyond a limited distance, transparency window vanishes. So far, there is no report on the investigation of the coupling distance of different coupling mechanisms. In this paper, we tailor transmission window of EIT with two different coupling mechanisms. One coupling way is electric resonance near field coupling to magnetic resonance, and the other way is simultaneous electric resonance and magnetic resonance. The two coupling mechanisms is belong to near field subwavelength scale coupling. The coupling based on electric resonance near field coupling to magnetic resonance is caused by incident electric field. For simultaneous electric resonance and magnetic resonance, not only incident electric field but also the incident magnetic field pays an important role on the near field coupling. The role of incident magnetic field has an import influence on the susceptibility of the system as shown in Eq. 8. Therefore, the influence of incident magnetic field based on simultaneous electric resonance and magnetic resonance leads to relatively smaller susceptibility change compared with that based on electric resonance near field coupling to magnetic resonance when the coupling distance changes. The relatively smaller susceptibility change causes relatively smaller EIT transmission window change compared with that based on electric resonance near field coupling to magnetic resonance. The simulated results show that EIT transmission window based on simultaneous electric resonance and magnetic resonance is still visible at large coupling distance, however, EIT transmission window based on electric resonance near field coupling to magnetic resonance vanishes. Hence, EIT based on simultaneous electric resonance and magnetic resonance has a larger coupling distance compared with that based on electric resonance near field coupling to magnetic resonance. The relatively large coupling distance is due to the relatively small susceptibility change based on the simultaneous electric resonance and magnetic resonance.

## Results

**Figure 1:**
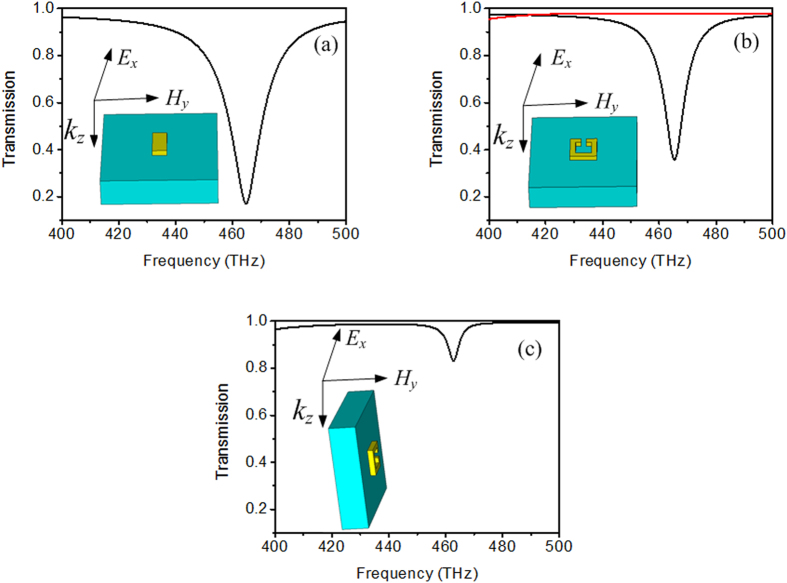
(**a**) Simulated transmission spectra of cut wire. (**b**) Simulated transmission spectra of SRR (red line) when the polarizations of electric field and magnetic field are along *x* axis and *y* axis, respectively. Simulated transmission spectra of SRR (black line) when the polarizations of electric field and magnetic field are along *y* axis and *x* axis, respectively. (**c**) Simulated transmission spectra of SRR when the magnetic field is perpendicular to the surface of SRR.

Figure 1(a,b) show the simulated transmission spectra of the cut wire and split ring resonator (SRR), respectively. The materials of the cut wire and the SRR are selected as gold. The permittivity of the gold is modeled as the Drude mode,


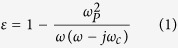


where the plasma frequency (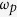) and the collision frequency (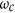) are 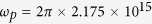 rad/s and 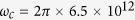 rad/s 31, respectively. The substrate is glass with the dissipation factor of 0.0001 and the index of refraction of 1.55. When the polarization of electric field is along x axis, as depicted by the inset of Fig. 1(a), there is a transmission dip at transmission spectra and the electric resonator is excited by incident electric field. For the SRR, when the polarizations of incident electric field and magnetic field are along *y* and *x* axis, respectively, there exists a transmission dip at transmission spectra, as depicted in Fig. 1(b). However, when the polarizations of electric field and magnetic field are illustrated as in Fig. 1(a), there is no transmission dip at transmission spectra as depicted in red line in Fig. 1(b). It conforms that SRR cannot be directly excited by incident electromagnetic wave with polarization as described in Fig. 1(a). When the polarization of magnetic field is perpendicular to the surface of SRR, there is a transmission dip at transmission spectra and the magnetic resonator is excited by incident magnetic field, as depicted in Fig. 1 (c).

**Figure 2:**
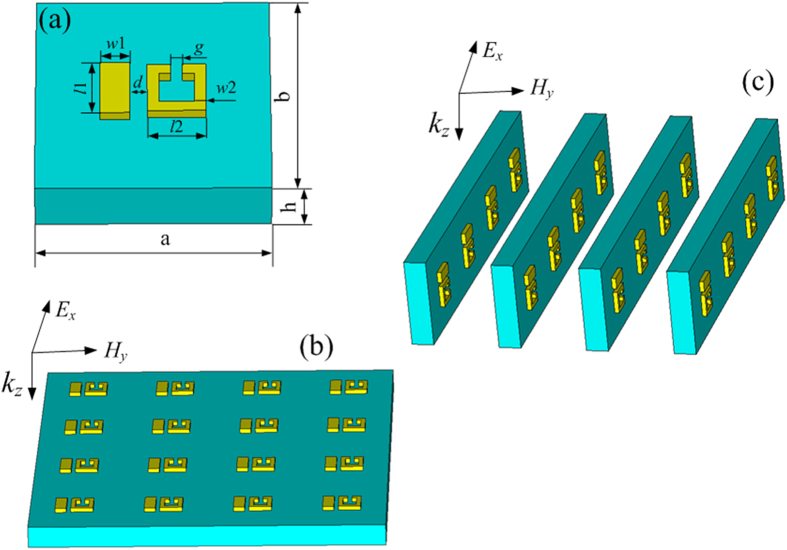
(**a**) Schematic view of EIT unit structure consisting of the cut wire, SRR and the glass. (**b**) Schematic view of EIT with electric resonance near field coupling to magnetic resonance. (**c**) Schematic view of EIT with simultaneous electric resonance and magnetic resonance. The geometrical parameters are as follows: *a* = 400 nm, *b* = 400 nm, *l*1 = 108 nm, *w*1 = 50 nm, *l*2 = 100 nm, *w*2 = 20 nm, *h* = 100 nm and *g* = 20 nm.

Figure 2(a) depicts the artificially united structure of EIT consisting of the cut wire, SRR and the glass. The coupling distance between the cut wire and SRR, *d*, is 25 nm and the thicknesses of the cut wire and SRR, *t*, are 20 nm. The rest geometrical parameters are as follows: *a* = 400 nm, *b* = 400 nm, *l*1 = 108 nm, *w*1 = 50 nm, *l*2 = 100 nm, *w*2 = 20 nm, *h* = 100 nm and *g* = 20 nm. As the cut wire and SRR are coupling with different mechanisms, the transmission properties of EIT structures are different. When the coupling mechanism of the cut wire and SRR is the electric resonance near field coupling to the magnetic resonance, the structure of EIT is described in Fig. 2(b). The distributions of the components of electric field and magnetic field can make sure that the cut wire can be excited by incident electric field and SRR cannot be excited, which is electric resonance near field coupling to magnetic resonance. The cut wire can be defined as bright mode and SRR as dark mode. When the coupling mechanism of the cut wire and SRR is simultaneous electric resonance and magnetic resonance, the structure of EIT is described in Fig. 2(c). The distributions of the components of electric field and magnetic field can make sure that the cut wire can be excited by incident electric field and SRR can be excited by incident magnetic field, which refers simultaneous electric resonance and magnetic resonance. The cut wire is bright mode and SRR is quasi-dark mode. The structure exhibiting EIT when excited by an incident plane wave polarized as in Fig. 2(c) resembles typical Huygens’ metasurfaces^32^,^33^,^34^. However, we believe that it is not a Huygens’ metasurface due to the following reasons: first, when Huygens’ metasurface is generated, the phase-shift needs to pi, however, the phase-shift of EIT transmission window can not reach to pi, and second, Huygens’ metasurface does not have slow light effect, however, EIT structure shown in Fig. 2(c) has slow light effect (see supplementary material).

**Figure 3:**
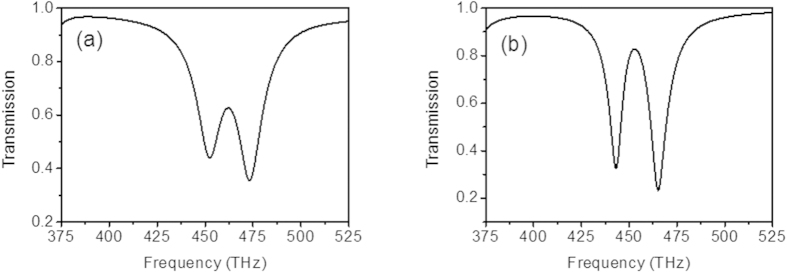
(**a**) Simulated transmission spectra of EIT with electric resonance near field coupling to magnetic resonance. (**b**) Simulated transmission spectra of EIT with simultaneous electric resonance and magnetic resonance.

When the coupling distance between the cut wire and SRR, *d*, is 25 nm, two EIT can exhibit visible transmission window as shown in Fig. 3(a,b). The characteristic of EIT transmission window is a transmission peak locates between two transmission dips. Both of the transmission windows of EIT are caused by the destructive interference of the cut wire and SRR as demonstrated in^12^. There is a little different resonance frequencies between Fig. 3(a,b), which is caused by the different SRR resonance frequencies in Fig. 1(b,c). When the cut wire and SRR have the same resonance frequencies as shown in Fig. 1(a,b), EIT transmission peak is located at the original transmission dip of Fig. 1(a,b) as shown in Fig. 3(a). SRR in Fig. 1(b,c) have different resonance frequencies. Therefore, it causes the different resonance frequencies in Fig. 3(a,b). One can see that EIT transmission window based on electric resonance near field coupling to magnetic resonance is less visible than that based on simultaneous electric resonance and magnetic resonance. It is due to the real part of susceptibility based on simultaneous electric resonance and magnetic resonance is more close to zero than that based on electric resonance near field coupling to magnetic resonance when the coupling distance is 25 nm.

**Figure 4:**
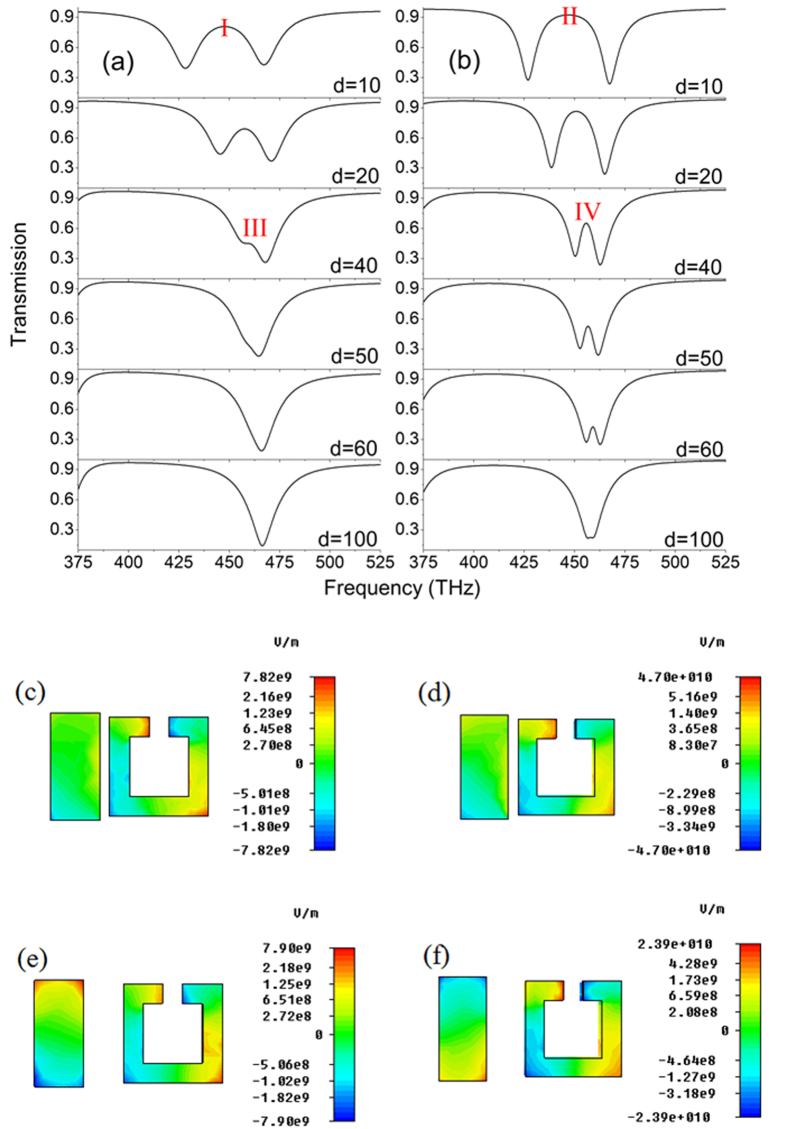
(**a**) Simulated EIT transmission spectrum based on electric resonance near field coupling to magnetic resonance with different coupling distances. (**b**) Simulated EIT transmission spectrum based on simultaneous electric resonance and magnetic resonance with different coupling distances. (**c**) The *z*-component of electric field distributions of EIT at I. (**d**) The *y*-component of electric field distributions of EIT at II. (**e**) The *z*-component of electric field distributions of EIT at III. (**f**) The *y*-component of electric field distributions of EIT at IV.

The coupling distance plays a crucial role in tailoring EIT transmission window. When d equals to 10 nm, two visible transmission windows can be observed as shown in Fig. 4(a,b). As d increases, both of the visible transmission windows deteriorate. When d equals to 40 nm, EIT transmission window based on electric resonance near field coupling to magnetic resonance vanishes, however, EIT transmission window based on simultaneous electric resonance and magnetic resonance still visible. As d further increases, EIT transmission window based on simultaneous electric resonance and magnetic resonance further deteriorates. When d equals to 100 nm, EIT transmission window based on simultaneous electric resonance and magnetic resonance also vanishes. Therefore, EIT based on simultaneous electric resonance and magnetic resonance has larger coupling distance compared with that based on electric resonance near field coupling to magnetic resonance. For EIT based on electric resonance near field coupling to magnetic resonance, the linearly coupled Lorentz oscillator mode can be expressed as follow:


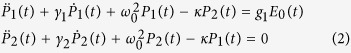


where 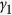 and 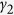 are the damping factors of 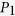 and 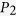, and 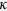 is the coupling coefficient between the two dipoles, and 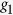 is the coupling coefficient between the electric dipoles and the incident electric field. Considering


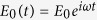



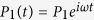



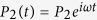


Then, the magnitude of the electric dipole 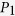 can be obtained:


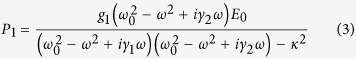


The susceptibility of the system can be determined by 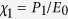. Using the second-order approximation, the susceptibility can be written as:


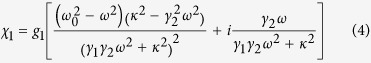


which is the same as shown in^12^. For simultaneous electric resonance and magnetic resonance, the linearly coupled Lorentz oscillator mode can be expressed as follow:


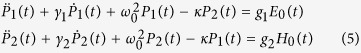


where 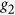 is the coupling coefficient between the magnetic dipoles and the incident magnetic field. Considering


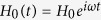


Then, the magnitude of the dipole 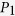 can be obtained:


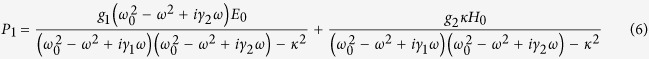


According to Eq. (6), it is obvious that the first term and the second term of Eq. (6) are respectively caused by incident electric and magnetic field.


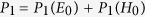



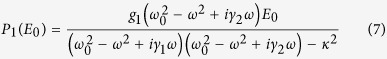



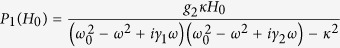


The susceptibility of the system can be determined by 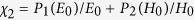. Using the second-order approximation, the susceptibility can be written as:


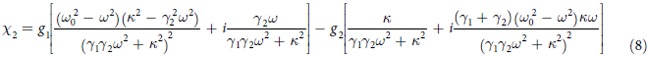


It is obvious that the first term of Eq. (8) is equal to Eq. (4). Therefore, 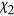 can be expressed as:


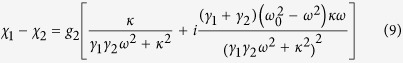


The derivation of the real parts of Eq. (9) can be written as:


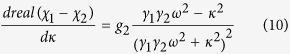


When 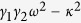 > 0, the slope of the real part of 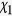 is larger than the slope of the real part of 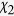, which indicates that EIT based on electric resonance near field coupling to magnetic resonance has larger susceptibility change compared with that based on simultaneous electric resonance and magnetic resonance when 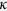 changes. The relatively large susceptibility change causes the relatively large EIT transmission window change. When d = 10 as shown in Fig. 4, there are visible transmission EIT window based on two coupling mechanisms and the real part of 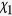 and 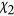 is equal to 0. When d = 40, the change of 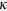 have larger influence on 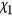. Therefore, EIT transmission window based on electric resonance near field coupling to magnetic resonance is not visible. However, EIT transmission window based on simultaneous electric resonance and magnetic resonance is still visible due to relative small change of 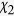.

When 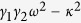 = 0, the slope of the real part of 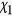 is equal to the slope of the real part of 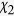, which indicates that EIT transmission window based on electric resonance near field coupling to magnetic resonance has the same change compared with that based on simultaneous electric resonance and magnetic resonance when 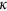 changes.

When 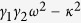 < 0, the slope of the real part of 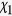 is smaller than the slope of the real part of 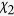, which indicates that EIT transmission window based on electric resonance near field coupling to magnetic resonance has smaller change compared with that based on simultaneous electric resonance and magnetic resonance when 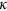 changes.

The electric field distributions of EIT at I, II, III, IV are depicted in Fig. 4(c–f), which is obtained by scattering a plane wave off a single cell with periodic boundary conditions. The incident plane wave in Fig. 4(c–f) is normal to structure and the power amplitude of incident plane wave is 0.5 watt. The polarization in Fig. 4(c,e) is the same as shown in Fig. 2(b) and the polarization in Fig. 4(d,f) is the same as shown in Fig. 2(c). For EIT based on electric resonance near field coupling to magnetic resonance, the electric field around SRR only comes from the near field coupling of the cut wire. However, for EIT based on simultaneous electric resonance and magnetic resonance, the electric field around SRR comes from two channels. One channel is the near field coupling of the cut wire. The other channel is the magnetic resonance directly excited by incident magnetic field. Therefore, there is more electric field around SRR for EIT based on simultaneous electric resonance and magnetic resonance as shown in Fig. 4(d,f), which leads to large coupling distance. Figure 4(c,e) depict the z-component of electric field distributions of EIT based on electric resonance near field coupling to magnetic resonance at I and III. Figure 4(d,f) depict the y-component of electric field distributions of EIT based on simultaneous electric resonance and magnetic resonance at II and IV. From Fig. 4(c,d), we can see that most of electric field distribute around SRR and there is few electric field distributed along the cut wire, which causes the transmission peaks at I and II. For Fig. 4(e,f), the electric field distributions around SRR and along the cut wire are different. The electric field distributions around SRR are same and the electric field distributions along the cut wire are out of phase, which leads to the transmission dip at III and the transmission peak at IV.

## Discussion

In conclusion, the coupling distance between the cut wire and SRR has a crucial role in determining the transmission window. In order to obtain relatively large coupling distance, two kind coupling mechanisms of EIT have been compared. The results show that EIT based on simultaneous electric resonance and magnetic resonance has larger coupling distance compared with that based on electric resonance near field coupling to magnetic resonance. The relatively large coupling distance is due to the relatively small susceptibility change based on the simultaneous electric resonance and magnetic resonance.

## Methods

In the simulations, the materials of the cut wire and the SRR are selected as gold. The permittivity of the gold is modeled as the Drude mode, where the plasma frequency (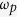) and the collision frequency (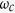) are 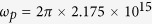 rad/s and 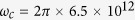 rad/s 31, respectively. The simulated results are carried out using the commercial finite difference time domain (FDTD) software package (CST Microwave Studio).

## Additional Information

**How to cite this article**: Li, H.-m. *et al.* Tailoring electromagnetically induced transparency with different coupling mechanisms. *Sci. Rep.* **6**, 21457; doi: 10.1038/srep21457 (2016).

## Supplementary Material

Supplementary Information
