# Supplementary material for: Tailoring electromagnetically induced transparency with different coupling mechanisms
Source: Sci Rep. 2016 Feb 22;6:21457. doi: 10.1038/srep21457 (PMC4761887; doi:10.1038/srep21457)
Supplement: Supplementary Information [file srep21457-s1.pdf]

Tailoring electromagnetically induced transparency with different coupling mechanisms

Hai-ming Li,[1,*] Shao-bin Liu,[1,*] Shen-yun Wang,[2] Si-yuan Liu,[1] Yan Hu,[1] and Hai-bin Li[3]

[1]Key Laboratory of Radar Imaging and Microwave Photonics, Nanjing University of Aeronautics and Astronautics, Nanjing, 210016, China.

[2]Research Center of Applied Electromagnetics, Nanjing University of Information Science & Technology, Nanjing, 210044, China.

[3]College of Materials and Chemical Engineering, Anhui Jianzhu University, Hefei 230022, China

[*]Correspondence and requests for materials should be addressed to H. L. (email: lhmm24@163.com) or S. L. (email: lsb@nuaa.edu.cn)

Here, we illustrate the difference between EIT structure based on the simultaneous electric resonance and magnetic resonance (shown in Fig. 2(c)) and the Huygens' metasurface. Huygens' metasurface can beam shaping, steering and focusing capabilities, however, it can not slow down light or have slow light effect. In addition, when it is generated, the phase-shift needs to pi. When the coupling distance d=10 nm, the degree plot of EIT structure based on the simultaneous electric resonance and magnetic resonance is depicted in Fig. 1s. We can see that the phase-shift of EIT transmission window (shaded area) can not reach to pi. Therefore, we believe it is not Huygens' metasurface.

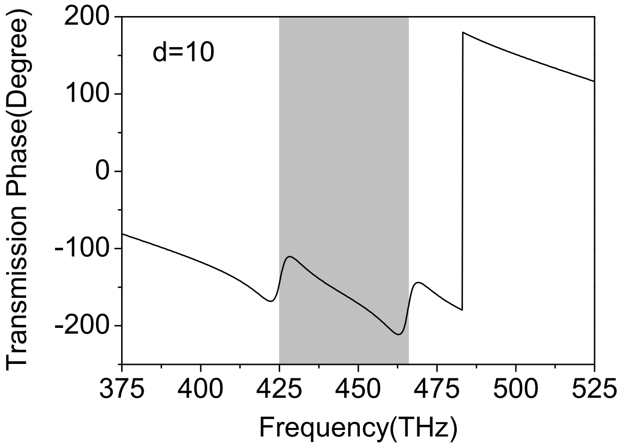

FIG. 1s. Simulated transmission phase of EIT with d=10 nm based on the simultaneous electric resonance and magnetic resonance.

The group index plot of EIT based on the simultaneous electric resonance and magnetic resonance is also depicted in Fig. 2s when the coupling distance *d*=10 nm. We can see that the maximum value of group index can reach to 8 within the EIT transmission window (shaded area), which confirms EIT based on the simultaneous electric resonance and magnetic resonance has slow light effect. Therefore, we believe it is not Huygens' metasurface.

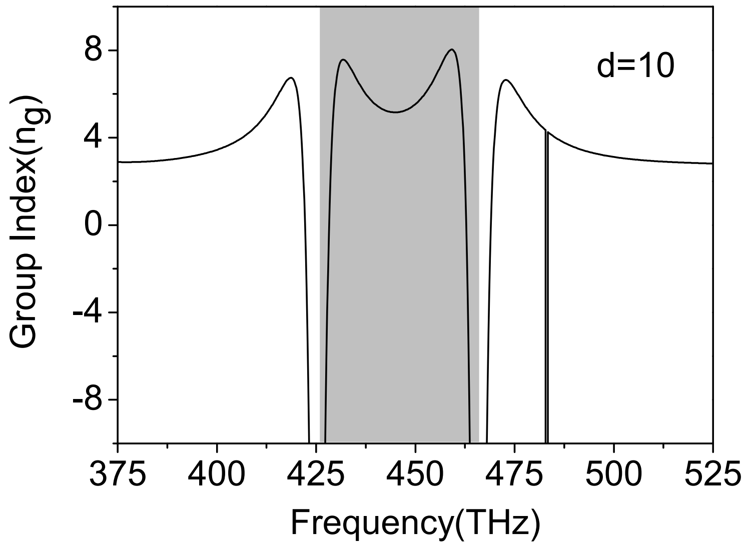

FIG. 2s. Simulated group index of EIT with *d*=10 nm based on the simultaneous electric resonance and magnetic resonance.
